# Ogilvie syndrome after use of vincristine: tomographic findings

**DOI:** 10.1590/0100-3984.2015.0162

**Published:** 2017

**Authors:** Fernanda Miraldi Clemente Pessôa, Leonardo Kayat Bittencourt, Alessandro Severo Alves de Melo

**Affiliations:** 1 Hospital Universitário Antonio Pedro - Universidade Federal Fluminense (HUAP-UFF), Niterói, RJ, Brazil.; 2 Universidade Federal Fluminense (UFF), Niterói, RJ, Brazil.

**Figure 1:**
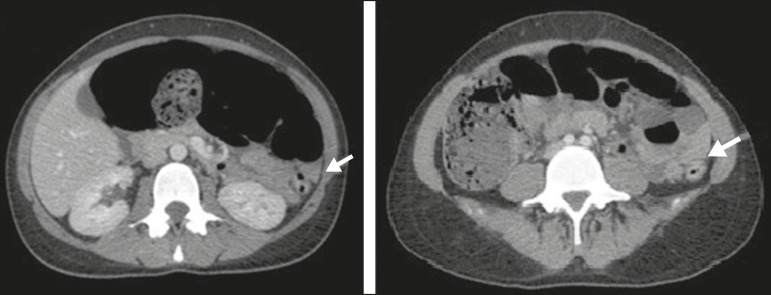
CT scan of the abdomen, in axial sections, obtained 60 s after injection of iodinated anionic contrast. Note the intestinal obstruction at the level of the proximal descending colon, adjacent to the splenic flexure. Distension of the transverse colon, ascending colon, and cecum, with the presence of fecal matter. The transitional zone can be seen at the level of the splenic flexure (arrow), with no evident obstructive material.

**Figure 2:**
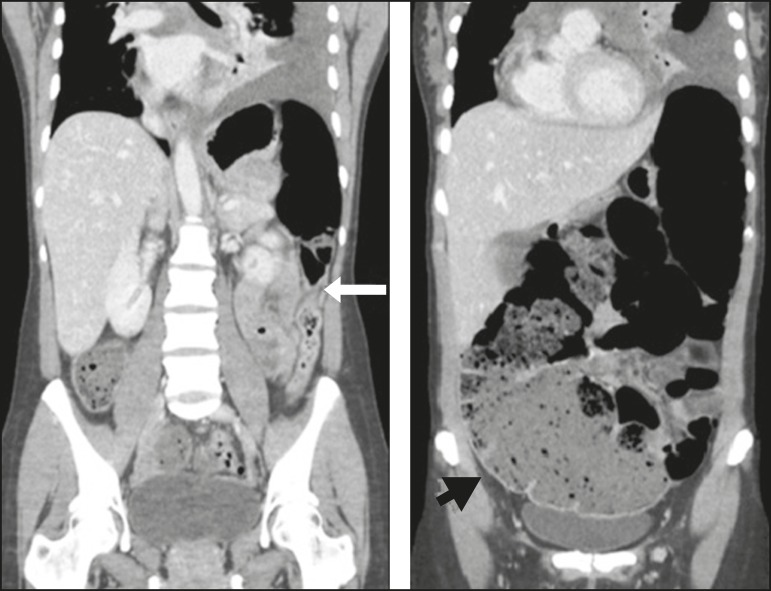
Coronal reconstruction of a CT scan, providing a better view of the transitional zone, where an abrupt transition to a normal caliber segment is observed, with no evident occlusive lesion (arrow). Note the marked dilation of the cecum, which measured 14 cm in diameter (arrowhead). Left pleural effusion can also be seen.

Dear Editor,

A 33-year-old female patient with diffuse large B-cell non-Hodgkin lymphoma was evaluated two days after the end of the first cycle of chemotherapy. The chemotherapy regimen comprised a five-day cycle, including rituximab, cyclophosphamide, doxorubicin, vincristine, and prednisone on the first day, whereas prednisone alone was administered on the four remaining days. She reported left pleuritic pain and flatus with evacuation. She was afebrile. The abdomen was flaccid and peristaltic, without painful decompression. Because she had neutropenia, she was hospitalized, after which she evolved to having no bowel movements, with the smell of feces on her breath and painful abdominal decompression. Computed tomography (CT) of the chest and abdomen showed left pleural effusion, intestinal obstruction in the descending colon adjacent to the splenic flexure, that segment being of normal caliber, without occlusive lesions, although the transverse ascending colon and cecum were dilated, the latter being 14 cm in diameter (Figures 1 and 2). There was gas in the rectal ampulla. These findings were suggestive of acute colonic pseudo-obstruction. Colonoscopic decompression and enema use were not considered because of the risk of cecal perforation. Therefore, the pseudo-obstruction was confirmed surgically. Thereafter, the patient was treated with gastric rest and her electrolyte levels were monitored.

Ogilvie’s syndrome was named after William Heneage Ogilvie, who, in 1948, described a disorder of gastrointestinal motility, with dilation of the cecum and right colon in the absence of mechanical obstruction, that was autonomic in origin, with suppression of parasympathetic activity and activation of sympathetic activity^(1)^.

The acute form of Ogilvie’s syndrome arises from an autonomic imbalance, with a mismatch between parasympathetic and sympathetic activity, which are downregulated and upregulated, respectively. The distal colon is often atonic, whereas the proximal colon can still be functional^(2)^. Some chemotherapeutic agents have been implicated, such as those in the rituximab-cyclophosphamide-doxorubicin-vincristine-prednisone regimen, as have factors such as trauma, acute myocardial injury, electrolyte disturbances, hypothyroidism, renal failure, and neuropathy^(2)^. Lee et al.^(3)^ observed that cancer patients developed Ogilvie’s syndrome two to ten days after infusion of vincristine, the syndrome resolving after its discontinuation. Sandler et al.^(4)^ found that patients treated with vincristine experienced abdominal pain and constipation within the first 4-72 hours after receiving the drug. Neutropenia and the use of antibiotic therapy have also been implicated in the development of the syndrome^(3)^.

The symptoms of Ogilvie’s syndrome include abdominal distension, abdominal pain, vomiting of fecal matter, and constipation^(1,5)^. Signs of peritonitis can indicate cecal perforation with pneumoperitoneum^(6)^, especially when the distension is greater than 12 cm and lasts for more than six days. For evaluating diseases of the colon, CT has been shown to be the method of choice^(7-11)^. In Ogilvie’s syndrome, CT is a useful for identifying the obstruction and determining the underlying cause^(12)^, the main findings being dilation extending from the cecum to the transverse colon, with a transition zone in the splenic flexure, where the caliber of the adjoining loop is considerably smaller. The treatment involves the use of parasympathomimetic agents that increase colonic motility^(13)^, endoscopic decompression or right hemicolectomy, the last being required in the presence of cecal ischemia or perforation.

Colonic pseudo-obstruction is associated with the use of chemotherapy. It is characterized by dilation of the loops of the colon and transitional zone. Attention should be paid to signs of perforation and the risk of death from cecal rupture.
